# Kritische Ereignisse in der Akutschmerztherapie – eine Risikoanalyse von CIRS-Meldungen

**DOI:** 10.1007/s00101-021-01041-3

**Published:** 2021-10-06

**Authors:** J. Erlenwein, M. Maring, M. I. Emons, H. J. Gerbershagen, R. M. Waeschle, L. Saager, F. Petzke

**Affiliations:** 1 Klinik für Anästhesiologie, Universitätsmedizin Göttingen, Robert-Koch-Str. 40, 37075 Göttingen, Deutschland; 2 Klinik für Anästhesiologie, Operative Intensivmedizin, Notfallmedizin und Schmerztherapie, Marienhospital, Gelsenkirchen, Deutschland

**Keywords:** Zwischenfälle, Schmerzdienst, Patientenkontrollierte Analgesie, Peridurale Analgesie, Opioide, Critical incidents, Pain service, Patient controlled analgesia, Peridural analgesia, Opioids

## Abstract

**Hintergrund:**

Tätigkeitsgebiete mit vielen Schnittstellen, wie die Akutschmerztherapie, gelten per se als Bereiche, in denen ein erhöhtes Risiko für Fehler und Zwischenfälle besteht.

**Ziel der Arbeit:**

Ziele waren die Risikoidentifikation und Graduierung des Risikos von gemeldeten Zwischenfällen im Kontext der Akutschmerztherapie.

**Material und Methoden:**

Aus 5365 Fällen des bundesweiten Meldesystems CIRSmedical Anästhesiologie wurden 508 Berichte mit dem Selektionskriterium „Schmerz“ identifiziert und 281 Berichte (55 %) analysiert und anhand einer Risikomatrix graduiert.

**Ergebnisse:**

Diese eingeschlossenen Fälle standen im Kontext parenteraler Analgetikaapplikationen (40 %) und rückenmarknaher (40 %) bzw. peripherer Regionalanästhesieverfahren (23 %) sowie der patientenkontrollierten Analgesie in 13 % der Meldungen (Mehrfachnennung möglich). Die meisten Ereignisse waren anhand der Schilderungen auf fachliche Fehler, Kommunikationsdefizite und ein Abweichen von der Routine zurückzuführen. Sie basierten meist auf Zugangs‑, Dosis- oder Wirkstoffverwechslungen. Etwa ein Drittel der Fehlerquellen war organisatorischer Art. Ein mögliches vitales Risiko war anhand der Berichte in 59 % der Fälle anzunehmen; 16 % der Fälle gingen mit tatsächlichen vitalen Komplikationen einher. Die Risikograduierung ergab zusammengefasst in Risikoklassen in 7 % ein „extrem hohes“, in 62 % ein „hohes“, in 25 % ein „moderates“ und in 6 % ein „niedriges“ Risiko.

**Diskussion:**

Insgesamt stellte sich ein relevantes Risikopotenzial für die Patienten dar. Gerade Zwischenfälle mit menschlichen Fehlern, Abweichen von der Routine und organisatorischen Aspekten gehen mit hohem Risiko einher.

## Abstract (en)

**Background:**

Areas of activity with many intersections pose an increased risk for errors and critical incidents. Therefore, procedures for acute pain therapy are potentially associated with an increased risk for adverse patient outcomes.

**Objective:**

The aim was to identify and grade the risk of critical incidents in the context of acute pain management.

**Material and methods:**

The register of the nationwide reporting system critical incident reporting system of the Professional Association of German Anesthesiologists, the German Society for Anesthesiology and Intensive Care Medicine and the Medical Center for Quality in Medicine (CIRSmedical Anesthesiology) was screened for incidents concerning pain management. Out of 5365 cases reported nationwide up to 24 March 2020, 508 reports with the selection criterion “pain” could be identified and reviewed and 281 reports (55%) were included in a systematic analysis.

**Results:**

Of the 281 reports most came from anesthesiology departments (94%; 3% from surgery departments and 3% from other departments). The reported cases occurred most frequently on normal wards but a relevant proportion of the reports concerned intermediate and intensive care units or areas covered by a pain service (PS). Based on the description of the incident in the report, an involvement of the PS could be assumed for 42% of the cases. In terms of time, most of the events could be assigned to normal working hours (90%) and working days (84%; weekends 16%).

The analyzed reports related to parenteral administration of analgesics (40%) and central (40%) or peripheral regional anesthesia procedures (23%) and 13% of the reports related to patient-controlled intravenous analgesia (PCIA; multiple answers possible). Most of the events were caused by technical errors, communication deficits and deviations from routine protocols. A relevant number of the cases were based on mix-ups in the administration route, the dosage, or the active agent. About one third of the sources of error were of an organizational nature, 59% of the cases posed a possible vital risk and in 16% of cases patients had vital complications. The risk grading by risk matrix resulted in an extremely high risk in 7%, a high risk in 62%, a moderate risk in 25% and a low risk in 6% of the cases.

Comparing risk assessment of events with involvement of different analgesic methods, multiple medication, combination of analgesic methods or involvement of PS showed no significant differences. Likewise, no differences could be identified between the risk assessments of events at different superordinate cause levels. If more than one overriding cause of error had an impact, initially no higher risk profile was found.

**Conclusion:**

Incidents in the context of acute pain management can pose high risks for patients. Incidents or near-incidents are mostly related to mistakes and lack of skills of the staff, often due to time pressure and workload as well as to inadequate organization.

## Hintergrund und Fragestellung

„Krankenhäuser sind gefährliche Orte …!“ Mit dieser Aussage fasst Wocher das Risiko für Patienten, welches unabhängig von der Erkrankung allein durch den Krankenhausaufenthalt besteht, zusammen [35]. Risiken und Zwischenfälle, die sich aus dem Behandlungsprozess ergeben, haben nicht nur für die Patienten (und Behandler) negative Folgen. Durch ungünstige Verläufe, Komplikationen oder Schadensereignisse kommt es auch aufseiten der Kliniken durch verlängerte Behandlungs- und Liegezeiten, Revisionseingriffe, Rechtsstreitigkeiten und ggf. Schadensersatz zu Mehrkosten und Erlösdefiziten [18, 23, 35, 36].

Besonders im Kontext schnittstellenreicher Behandlungsprozesse ergibt sich ein hohes Risiko für unerwünschte Ereignisse [14]. Ein solcher Prozess ist die Behandlung akuter Schmerzen. An der Akutschmerztherapie sind als interdisziplinäres und interprofessionelles Tätigkeitsfeld zahlreiche Akteure beteiligt, sodass hier ein erhöhtes Risikoprofil vorliegt [25, 28]. Der Einsatz von programmierbaren Pumpen, gemischten Medikamentenansätzen und oraler oder parenteraler (patientengesteuerter) Opioidapplikation birgt zusätzliche, potenzielle Risiken [1, 17, 19, 27, 33, 34]. Das Thema systematischer Risikoanalysen und -steuerung wurde für den Bereich der Akutschmerztherapie bisher nur wenig betrachtet. Neben der reinen Analyse der Komplikationshäufigkeit gibt es bisher nur wenige Untersuchungen, die systemische Ansätze zu Risikoidentifikation, -bewertung oder -reduktion nutzen [1, 28, 33]. Ein etabliertes Instrument zur Identifikation von Risiken ist das Critical Incident Reporting System (CIRS). Die Methode nutzt die Ressource beobachteter Ereignisse durch meist anonymisierte Meldungen [16].

Ziele der folgenden Arbeit waren die inhaltliche Analyse, die Risikoidentifikation und die Graduierung des Risikos von Zwischenfällen im Kontext der Behandlung akuter Schmerzen, die im bundesweiten frei zugänglichen Meldesystem CIRSmedical Anästhesiologie gemeldet wurden. Quantitative Aussagen über die Gesamtheit aller Zwischenfälle der Akutschmerztherapie können methodologisch nicht gemacht werden, sondern nur die eingeschlossenen Fallberichte charakterisiert werden. Zusätzlich wurde ein Vergleich der in der Analyse getroffenen Risikoklassifikation sowohl mit Merkmalen des Analgesieverfahrens als auch der einwirkenden Fehlerursachen durchgeführt. Hier war es Ziel darzustellen, welche Aspekte im Kontext des klinischen Risikomanagements niedrige oder hohe Risikopotenziale aufweisen könnten.

## Studiendesign und Untersuchungsmethoden

### Selektion von Fallberichten

Der Berufsverband Deutscher Anästhesisten (BDA), die Deutsche Gesellschaft für Anästhesiologie und Intensivmedizin (DGAI) und das Ärztliche Zentrum für Qualität in der Medizin (ÄZQ) betreiben seit dem 01.04.2010 ein bundesweites Ereignismeldesystem (CIRSmedical Anästhesiologie, CIRS-AINS – www.cirs-ains.de), über welches Ereignisse, Zwischenfälle und Komplikationen (mit und ohne Patientenschaden) anonym gemeldet und standardisiert erfasst werden können. Alle bis zum Stichtag 24.03.2020 gemeldeten und online einsehbaren Fälle mit dem Kriterium „Schmerz“ wurden über die Suchmaske identifiziert. Von 5365 aufrufbaren Fällen wurden 508 Fälle mit dem Selektionskriterium „Schmerz“ identifiziert. Diese Fälle wurden einzeln aufgerufen und die Dokumentation als PDF heruntergeladen. Bei der ersten Sichtung (M.M., J.E.) wurden Fälle, in denen es inhaltlich nicht um die Behandlung von akuten Schmerzen ging, ausgeschlossen. Außerdem wurden im Zuge dieser Sichtung die Auswertungskriterien abschließend definiert.

Nach Einzelbewertung wurden 281 Fallberichte (55 %), bei denen das berichtete Ereignis im Kontext der Akutschmerztherapie stand, eingeschlossen. Berücksichtigt wurden gemeldete Ereignisse von der perioperativen Anlage spezieller Analgesieverfahren bis zur Akutschmerzbehandlung im Aufwachraum, auf der Intensivstation, der Normalstation und in Funktionsbereichen. Die im Ergebnisteil dargestellten Inhalte beziehen sich auf diese aus CIRS-AINS eingeschlossenen Fälle.

### Inhaltliche Analyse

Es folgte die standardisierte Analyse (M.M.) der identifizierten Fälle anhand der zuvor definierten Auswertungskriterien, soweit sie anhand der vorgegebenen Dokumentation erfassbar waren. Es wurden zunächst Items erfasst, die durch die elektronische Eingabemaske des Meldesystems mit Auswahlantworten vorgegeben waren:

- Fachgebiet,
- Arbeitsbereich (berücksichtigt: Anästhesieeinleitung, OP/Anästhesieausleitung, Aufwachraum, Intensivstation/Intermediate-Care-Station, Schmerzdienst, Notfallteameinsatz, Normalstation, Funktionsbereich/Diagnostik, Kreißsaal, Sonstiges),
- Ereignistag (Wochentag, Wochenende),
- Versorgungsart (Routineversorgung, Notfallversorgung),
- American Society of Anesthesiologists Physical Status Classification System (ASA-PS I–V, Klassifikation zur Graduierung des präoperativen körperlichen Gesundheitszustandes von Patienten): ASA-PS I: normaler, gesunder Patient, ASA-PS II: Patient mit leichter Allgemeinerkrankung, ASA-PS III: Patient mit schwerer Allgemeinerkrankung, ASA-PS IV: Patient mit schwerer Allgemeinerkrankung, die eine ständige Lebensbedrohung darstellt, ASA-PS V: moribunder („totgeweihter“) Patient, der ohne Operation voraussichtlich nicht überleben wird),
- allgemeiner Patientenzustand (Freitext),
- subjektive Einschätzung der Häufigkeit des berichteten Ereignisses in der Institution durch den Meldenden/Berichterstatter („fast täglich“, „jede Woche“, „jeden Monat“, „mehrmals pro Jahr“, „selten“, „nur dieses Mal“),
- Berufsgruppe des Meldenden/des Berichterstatters (Arzt, Pflege, Sonstige),
- Berufserfahrung des Meldenden/Berichterstatters (bis 5 Jahre, > 5 Jahre).

Neben den durch die Eingabemaske vorgegebenen Kriterien wurden die Fälle standardisiert inhaltlich anhand der Freitextbeschreibung ausgewertet. Aufgrund der Anonymität der Fallberichte war eine Nachfrage bei den Berichtenden nicht möglich. Zur Minimierung eines Bewertungsbias wurde dabei eine einfache dichotome Bewertung bevorzugt. Definierte Auswertungskriterien im Kontext des Schmerzmanagements waren:

- Patientengruppe (Erwachsener, Kind, Schwangere),
- Angaben zur Schmerztherapie:
  - Analgesieverfahren, Applikationsweg (oral, parenteral, PCIA, peripheres oder zentrales regionalanästhesiologisches Verfahren, inkl. Katheteranlage; Mehrfachnennung möglich),
  - Substanzgruppen (Nichtopioide, Opioide, Koanalgetika, Sedativa, Ketamin, Clonidin, Lokalanästhetika; Mehrfachnennung möglich);
- Einordnung der Schmerzproblematik (allgemeine Routineanalgesie, komplexer Behandlungskontext);
- Beteiligung eines Schmerzdienstes (ja/nein).

Zudem wurden in der Analyse die Fälle anhand der zur Verfügung stehenden Informationen hinsichtlich potenzieller Folgen und Gefährdung der Patienten ausgewertet:

- Schmerzexazerbation als Folge des Ereignisses (ja/nein),
- Patientenbelastung/Unwohlsein im Rahmen des Ereignisses (ja/nein),
- berichtete vitale Komplikation^333^ (ja/nein),
- darüber hinausgehender Patientenschaden^444^ (ja/nein),
- Vitale Komplikation^1^/Patientenschaden^2^ wäre potenziell möglich gewesen (ja/nein).Bitte prüfen Sie die ethischen Richtlinien und bestätigen oder korrigieren.

Die Analyse der Fallberichte erfolgte, soweit das anhand des verfügbaren Kontextwissens der Berichte möglich war, angelehnt an eine Fehler-Ursachen-Analyse („root cause analysis“) [26]. Ziel war neben der Beschreibung der Problematik („Problemebene“) die Zuordnung zugrunde liegender Ursachen. Dabei wurde zwischen der unmittelbaren Ursachenebene der Ereignisse und einer übergeordneten Ursachenebene differenziert. Es konnten nach initialer Sichtung der Fallberichte und Entwicklung der Auswertungskriterien für die differenzierte Analyse die folgenden Aspekte in der jeweiligen Kategorie identifiziert und als Auswertungskriterien gruppiert werden:

- Auf der Problemebene konnten folgende Aspekte zusammenfassend identifiziert werden: Fehldosierung, falsche Konzentration der Substanz, Verwechslung der Substanz, falscher Applikationsweg, Patientenverwechslung, Handhabung, Fehllage (z. B. Katheter/i.v.-Zugang), paradoxe Reaktion auf Analgetika, rechtliche Problematik (z. B. Aufklärungszeitpunkt), Indikationsstellung, Lagerung, Medikamentennebenwirkung (Mehrfachnennung möglich).
- Auf Ebene unmittelbar zum Problem führender Ursachen (hier unmittelbare Ursachenebene) konnten folgende Aspekte zusammenfassend identifiziert werden: Abweichung von der Routine, Ähnlichkeit zu anderen Abläufen, Verfügbarkeit von Material/Personal, Ähnlichkeit der Ampullen mit unterschiedlichem Inhalt, fachlicher Fehler, fehlende Patienteninformationen (z. B. nichtbekannte Allergie), Materialfehler, Kommunikation zwischen Patient und Mitarbeiter, Kommunikation unter Mitarbeitern (Mehrfachnennung möglich).
- Auf übergeordneter Ursachenebene konnten folgende Aspekte zusammenfassend identifiziert werden: menschliches (Fehl‑)Verhalten [Patienten, Angehörige oder Personal], Technik, Organisation (Mehrfachnennung möglich); Kombination von übergeordneten Ursachenebenen.

## Inhaltliche Reevaluation

Nach der ersten inhaltlichen Analyse (M.M.) erfolgte zur Verbesserung der Auswertungsqualität und Objektivierung der Ergebnisse eine erneute unabhängige Durchsicht der aus CIRS-AINS eingeschlossenen Fälle und der Ergebnisse der ersten inhaltlichen Analyserunde. Dabei erfolgte die inhaltliche Reevaluation der ersten Analyse durch zuvor nichtinvolvierte Autoren (M.I.E., R.M.W., J.E.). Fälle mit zunächst unterschiedlicher Einschätzung wurden im Anschluss gemeinsam diskutiert und dann im Konsens bewertet. In diesem Schritt erfolgte außerdem die gemeinsame Risikoklassifikation aller Fälle. Diese erfolgte im Konsens anhand einer Risikomatrix (s. unten).

## Risikoklassifikation und Risikomatrix

**Figure 1:**
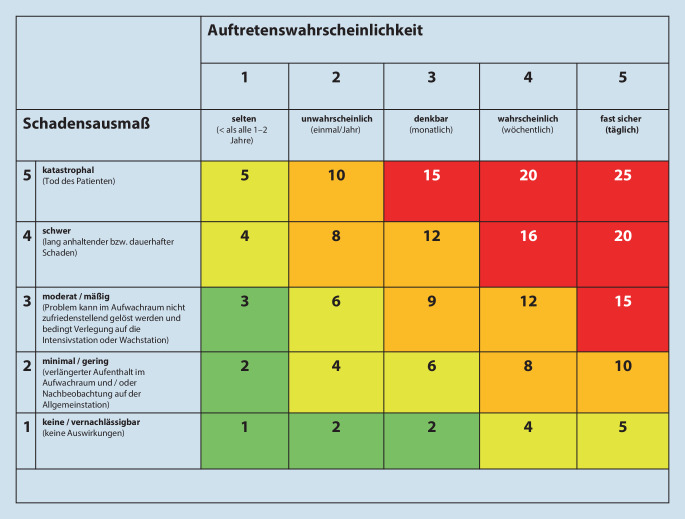

Im Rahmen der inhaltlichen Reevaluation wurden die Fälle anhand möglicher medizinischer Konsequenzen und Auftretenswahrscheinlichkeit in Risikogruppen klassifiziert (M.M., M.I.E., R.M.W., J.E.). Eine etablierte Methode zur Darstellung dieser Parameter ist die Risikomatrix [15, 20]. Bei der zweidimensionalen Darstellung wurden an der Abszisse die Auftretenswahrscheinlichkeit und an der Ordinate das mögliche Schadensausmaß eingeordnet (Abb. 1). Die Einstufung der Auftretenswahrscheinlichkeit erfolgte anhand der Variablen „selten“ (> 1 bis 2 Jahre) < „**unwahrscheinlich**“ (einmal/ Jahr) < „**denkbar**“ (monatlich) < „**wahrscheinlich**“ (wöchentlich) < „**fast sicher**“ (täglich), denen auf der Achse jeweils aufsteigend ein Wert von 1 bis 5 zugeordnet wurde. Das Schadensausmaß wurde anhand der Variablen „**keine/vernachlässigbar**“ (*keine Auswirkungen*) < „**minimal/gering**“ (*verlängerter Aufenthalt im Aufwachraum und/oder Nachbeobachtung auf der Allgemeinstation*) < „**moderat/mäßig**“ (*Problem kann im Aufwachraum nicht zufriedenstellend gelöst werden und bedingt Verlegung auf die Intensivstation oder Wachstation*) < „**schwer**“ (*lang anhaltender bzw. dauerhafter Schaden*) < „**katastrophal**“ (*Tod des Patienten*) klassifiziert [20]. Auch hier wurden jeweils aufsteigend Werte von 1 bis 5 zugeordnet.

Jeder Koordinate, die sich aus der Zuordnung von Auftretenswahrscheinlichkeit und dem möglichem Schadensausmaß ergab, wurde ein Wert per Multiplikation des jeweiligen Wertes von Auftretenswahrscheinlichkeit und Schadensausmaß zugeordnet (mögliche Werte minimal 1, maximal 25). Die Produkte wurden zur übersichtlichen Darstellung in die Risikoklassen „niedriges Risiko“ (Werte 1, 2, 3), „moderates Risiko“ (Werte 4, 5, 6), „hohes Risiko“ [8–10, 12] und „extrem hohes Risiko“ [15, 16, 19, 24] genutzt (Abb. 1). Die vergleichende statistische Auswertung erfolgte anhand der berechneten Produkte (Abb. 1; [20]).

## Statistische Analyse

Die Analyse und Darstellung dieser selektiven Stichprobe erfolgten primär deskriptiv mittels SPSS Statistics (Version 26 IBM, Armonk, USA). Der prozentuale Anteil wurde auf ganze Zahlen gerundet und bezog sich, wenn nicht anders beschrieben, auf die Gesamtzahl der eingeschlossenen Fälle. Ordinale Parameter wurden anhand des Medians (1. bis 3. Quartil) dargestellt. Die Merkmale „Analgesieverfahren“, „Kombination vs. keine Kombination von Analgesieverfahren“, „analgetische Mehrfachmedikation vs. keine Mehrfachmedikation“, „übergeordnete Ursachenebene“ (menschliches (Fehl‑)Verhalten, Technik, Organisation) sowie „eine vs. mindestens zwei einwirkende Fehlerursachen“ der 281 eingeschlossenen Fälle wurden statistisch anhand einer nichtparametrischen Testung hinsichtlich der Risikoklassifikation (Wert von Auftretenswahrscheinlichkeit multipliziert mit dem Wert des Schadensausmaßes) verglichen (Kruskall-Wallis-Test bei mehreren Gruppen, zur Post-hoc-Analyse oder primär bei Gruppenvergleich zwischen zwei Gruppen mittels Mann-Whitney-U-Test, Signifikanzniveau *p* ≤ 0,05; es erfolgte keine Anpassung des Signifikanzniveaus wegen Mehrfachtestung).

## Ergebnisse

### Einschlüsse und Fallcharakteristika

Von den 281 eingeschlossenen Berichten stammten die meisten Berichte aus dem Fachbereich Anästhesiologie (94 %, *n* = 263); 3 % (*n* = 10) aus der Chirurgie und 3 % (*n* = 8) aus anderen Fachbereichen. Die Berichtenden waren in 81 % (*n* = 221) ärztliche und in 19 % (*n* = 50) nichtärztliche Mitarbeiter (9 fehlende Angaben). In 80 % (*n* = 209) hatten sie mehr als 5 Jahre Berufserfahrung (20 fehlende Angaben).

Die berichteten Fälle ereigneten sich laut den Angaben am häufigsten auf der Normalstation, aber auch zu einem relevanten Anteil in Überwachungsbereichen und im Versorgungsbereich des Schmerzdienstes (SD; Tab. 1). In 42 % (*n* = 117) der Fälle ist anhand der Beschreibung von einer Beteiligung des SD auszugehen.

**Table 1:** 

Innerklinischer Arbeitsbereich	%	*n*
Normalstation	30	82
OP-Saal/Anästhesieausleitung	23	63
Intensivstation/Intermediate-Care-Station	16	43
Aufwachraum	12	34
Akutschmerzdienst	13	34
Kreißsaal	3	9
Notfallteam	1	4
Funktionsbereiche	1	3
Sonstige	1	4

5 fehlende Angaben; Mehrfachnennung möglich

Zeitlich konnten die meisten Ereignisse der Regelarbeitszeit zugeordnet werden (90 %, *n* = 247; außerhalb der Regelarbeitszeit 10 % (*n* = 26) und an Werktagen (84 %, *n* = 221); Wochenenden 16 % (*n* = 41)); acht Fallberichte konnten zeitlich nicht eingeordnet werden. Bei 95 % (*n* = 266) der Patienten handelte es sich, soweit dies aus der Schilderung nachzuvollziehen war, meist um Erwachsene und vermutlich um ein Kollektiv mit einem höheren Anteil morbider Patienten (ASA I 20 % (*n* = 53), ASA-Gruppe II 41 % (*n* = 106), ASA III 35 % (*n* = 90), ASA IV 3 % (*n* = 8), ASA V 1 % (*n* = 1), 23 Patienten nicht klassifiziert). In 4 % (*n* = 11) der Fälle bezog sich das Ereignis auf Schwangere. Bei 13 % der Fälle handelte es sich um Fälle mit Patienten mit vorbestehenden chronischen Schmerzerkrankungen (5 %, *n* = 14) bzw. teils komplexeren medikamentösen schmerztherapeutischen Therapiekonzepten.

## Beschreibung der vorgekommenen analgetischen Verfahren

Die meisten der eingeschlossenen Berichte standen im Kontext parenteraler Analgetikaapplikation (40 %, *n* = 109) und rückenmarknaher (40 %, *n* = 110) bzw. peripherer Regionalanästhesieverfahren (23 %, *n* = 64); Ereignisse im Kontext der PCIA betrafen 13 % (*n* = 35) der Meldungen (Mehrfachnennung möglich). In 22 % (*n* = 62) der Fälle wurden die Verfahren in Kombination angewandt.

### Regionale Schmerzkatheter.

Die beteiligten Regionalanästhesieverfahren waren mit 94 % (*n* = 166) meist in Form von Katheterverfahren umgesetzt, nur selten als „Single-shot“-Verfahren (6 %, *n* = 8): Am häufigsten war unter den anatomisch zuordenbaren Katheterverfahren der Periduralkatheter (77 %, *n* = 103). Periphere Schmerzkatheterverfahren kamen seltener vor (interskalenärer Katheter 5 % (*n* = 7), Plexus-axillaris-Katheter 1 % (*n* = 2), Paravertebralkatheter 1 % (*n* = 1), Femoraliskatheter 6 % (*n* = 8), Ischiadikuskatheter 8 % (*n* = 10), Kombination aus Femoralis- und Ischiadikuskatheter 2 % (*n* = 3)). In 35 der Fälle mit Katheterverfahren konnte anhand der Beschreibung nicht auf die genaue Lokalisation des Katheters rückgeschlossen werden.

### Lokalanästhetika.

Von den 74 Berichten, in denen Lokalanästhetika identifiziert werden konnten, war Ropivacain die am häufigsten benannte Substanz (95 %, *n* = 70), gefolgt von Bupivacain (5 %, *n* = 4) und Prilocain (3 %, *n* = 2; Mehrfachnennung möglich). In 35 % (*n* = 99) der berichteten Fälle musste von dem Einsatz eines Lokalanästhetikums ausgegangen werden, ohne dass dies genauer benannt war.

### Opioidtherapie.

In 54 % (*n* = 151) der Fälle wurde explizit von einer Opioidtherapie berichtet. Die am häufigsten genannten Wirkstoffe waren Piritramid (57 %, *n* = 56) und Sufentanil (28 %, *n* = 28), weitere (Remifentanil 7 % (*n* = 7); Oxycodon 5 % (*n* = 5); Fentanyl 4 % (*n* = 4); andere 10 % (*n* = 9); Mehrfachnennung möglich). In 6 % dieser Fälle (*n* = 9) erfolgte eine Kombination mehrerer Opioide. Für 34 % (*n* = 51) der Fälle konnten die konkret verwendeten Opioid-Wirkstoffe nicht eruiert werden.

### Nichtopioidanalgetika und Koanalgetika.

Der Einsatz von Nichtopioidanalgetika wurde in 39 (14 %) der Fallberichte beschrieben. Wenn ein Wirkstoff nachvollziehbar war, war dies am häufigsten Metamizol (67 %, *n* = 20; Mehrfachantworten möglich), gefolgt von Paracetamol (23 %, *n* = 7), Ibuprofen (6 %, *n* = 2), Parecoxib (6 %, *n* = 2) und Diclofenac (3 %, *n* = 1). Selten wurde in den Fällen über Koanalgetika berichtet (Clonidin *n* = 7, Ketamin *n* = 5, Pregabalin *n* = 2, Mehrfachantworten möglich). Der Einsatz von Sedativa wurde für 21 % (*n* = 49) aller Fälle berichtet.

Eine Mehrfachmedikation (mehrere Analgetika, auch mehrere einer Wirkstoffgruppe) oder die Kombination unterschiedlicher analgetischer Verfahren (z. B. regionale und systemische Analgesie) wurde in 43 % (*n* = 115) bzw. 22 % (*n* = 62) der Berichte eingesetzt. Zusätzlich muss anhand der Fallbeschreibung davon ausgegangen werden, dass mindestens bei 10 % (*n* = 29) weiteren Fällen eine Mehrfachmedikation erfolgte, ohne dass diese benannt war.

## Ursachenanalyse

### Problematik.

Die unerwünschten Ereignisse der eingeschlossenen Fälle basierten meist auf falschen Applikationswegen und Fehldosierungen bzw. Medikamentenverwechslungen und Handhabungsfehlern (Problemebene; Tab. 2).

**Table 2:** 

Problemebene	%	*n*
Falscher medikamentöser Applikationsweg	25	71
Fehldosierung	14	39
Medikamentenverwechselung	14	38
Handhabungsfehler (technisch/organisatorisch)	14	38
Falsche Indikationsstellung	13	35
Falsche Medikamentenkonzentration	7	19
Fehllage des Analgesieverfahren	6	18
Medikamentennebenwirkung	3	9
Fehlende/falsche Aufklärung	2	6
Lagerung	2	4
Paradoxe Medikamentenreaktion	1	2
Patientenverwechselung	1	2
Sonstige	2	4

Mehrfachnennung möglich

### Ursachenebene.

Ursächlich standen fachliche Fehler/fehlende Kompetenz, Kommunikationsprobleme zwischen Mitarbeitern, Abweichen von der Routine und die optische Ähnlichkeit von Medikamenten/Ampullen im Vordergrund (Ursachenebene, Tab. 3).

**Table 3:** 

Ursachenebene	%	*n*
Fachliche Fehler/fehlende Kompetenz	31	87
Kommunikationsprobleme zwischen Mitarbeitern	17	48
Abweichen von der Routine	15	40
Optische Ähnlichkeit von Medikamenten/Ampullen	13	36
Ähnlichkeit zu anderen Abläufen	11	30
Materialfehler	10	28
Verfügbarkeit von Ressourcen (Material/Personal)	4	10
Fehlende Patienteninformationen	2	5
Kommunikation zwischen Patient und Mitarbeiter	2	4

Mehrfachnennung möglich

### Übergeordnete Ursachenebene.

Das berichtete Geschehen wurde in der überwiegenden Anzahl der eingeschlossenen Fälle (95 %, *n* = 260) in den Kontext menschlichen (Fehl‑)Verhaltens eingeordnet (Mehrfachnennung möglich). In einigen Fällen (6 %, *n* = 15) lag laut Beschreibung ein Fehlverhalten des Patienten vor und in einem Fall durch Angehörige (Anschluss des Periduralkatheters durch Angehörige an den zentralen Venenkatheter). In ca. einem Drittel der Zwischenfälle stand das unerwünschte Ereignis im Kontext organisatorischer Aspekte (31 %, *n* = 85) und in 23 % (*n* = 62) der Fälle mit technischen Problemen (Mehrfachnennungen). In 49 % (*n* = 145) der Fälle war der Schilderung nach vom Einwirken mehrerer übergeordneter Ursachen auszugehen.

Bei der differenzierten Betrachtung organisatorischer Aspekte waren dies Mehrfachbelastung und Zeitnot des Personals (37 %, *n* = 32), unbekannte/nichtbeherrschte Technik/Material/Medikamente (30 %, *n* = 26), fehlende Einarbeitung 17 % (*n* = 15), fehlende materielle oder technische Ausstattung (9 %, *n* = 8) oder ungeklärte Verantwortlichkeit (8 %, *n* = 7; Mehrfachnennung möglich).

## Folgen für die Patienten und die Einrichtung

Als Konsequenz für den Patienten waren in je 33 % der beschriebenen Fälle Befindlichkeitsstörungen (*n* = 92) und/oder eine Schmerzexazerbation (*n* = 93) als Folgen beschrieben. In 16 % (*n* = 44) der Ereignisse wurden konkrete vitale Komplikationen berichtet. Darüber hinaus ergab die retrospektive Fallanalyse in 59 % (*n* = 166) der Fälle die Einschätzung einer potenziell vitalen Gefährdung im Kontext des unerwünschten Ereignisses für die Patienten – unabhängig davon, ob es tatsächlich zu einer Gefährdung kam oder nicht (z. B. akzidentielle i.v.-Applikation eines Lokalanästhetikums, Konzentrations‑/Dosisfehler bei Opioidtherapie/PCIA, fehlendes Monitoring etc.).

Ein unerwünschtes Ereignis, dass eine relevante therapeutische Intervention notwendig machte/bzw. mit zusätzlicher Belastung durch therapeutische Folgen für den Patienten verbunden war, die über das unmittelbare Fallgeschehen andauern, wurde für 10 % (*n* = 27) der Fälle anhand der zur Verfügung stehenden Informationen angenommen (Infobox 1).

Die Meldenden konnten in der Berichtsmaske (potenzielle) eigene Rückschlüsse oder erfolgte Veränderungen für den Behandlungsprozess oder die Einrichtung beschreiben. Dies erfolgte mehr oder weniger detailliert in 91 % (*n* = 256) der Berichte, meist ohne dass immer klar war, ob es sich hier um die Erwartungen/Vorschläge des Mitarbeitenden handelte oder um tatsächliche Vorhaben oder gar vollzogene Änderungen der Einrichtung. Als Konsequenzen gaben 38 % (*n* = 107) der Meldenden an, dass sich in ihren Einrichtungen das Verhalten der Mitarbeiter änderte/ändern sollte – im Sinne einer Sensibilisierung für Fehler; 22 % (*n* = 62) gaben an, dass eine Verbesserung der Kommunikation umgesetzt wurde/werden sollte. In 17 % (*n* = 49) der Einrichtungen sollten organisatorische Abläufe und in 10 % (*n* = 27) die Beschriftung der Medikamente/Perfusorleitungen verbessert werden. Technische Anpassungen waren bei 8 % (*n* = 21) der Melder geplant (Mehrfachnennung möglich).

## Einschätzung der Häufigkeit berichteter Ereignisse

In der CIRS-AINS-Berichtsmaske werteten die Meldenden die Häufigkeit der unerwünschten Ereignisse in 24 % (*n* = 65) der eingeschlossenen Fälle als „nur einmalig“, in 36 % (*n* = 99) als „selten“ oder in 21 % (*n* = 58) als „mehrmals pro Jahr“, in jeweils 8 % häufiger als „jeden Monat“ (*n* = 23) bzw. „jede Woche“ (*n* = 22). In 3 % (*n* = 8) der Fälle wurde angegeben, dass sich die Ereignisse „fast täglich“ ereigneten (in 6 Berichten fehlte die Einschätzung der Meldenden). 61 % (*n* = 171) der Zwischenfälle wurden in unserer Analyse anhand der Schilderung der Meldenden als zufällige Ereignisse eingestuft. In der Beschreibung der restlichen Fälle (39 %; *n* = 110) lagen Hinweise (oder Aussagen) vor, die vermuten ließen, dass sich die bestehende Fallkonstellation wiederholt in der betroffenen Klinik ereignete und das bestehende Problem bekannt war.

## Risikobewertung anhand der Risikomatrix

**Figure 2:**
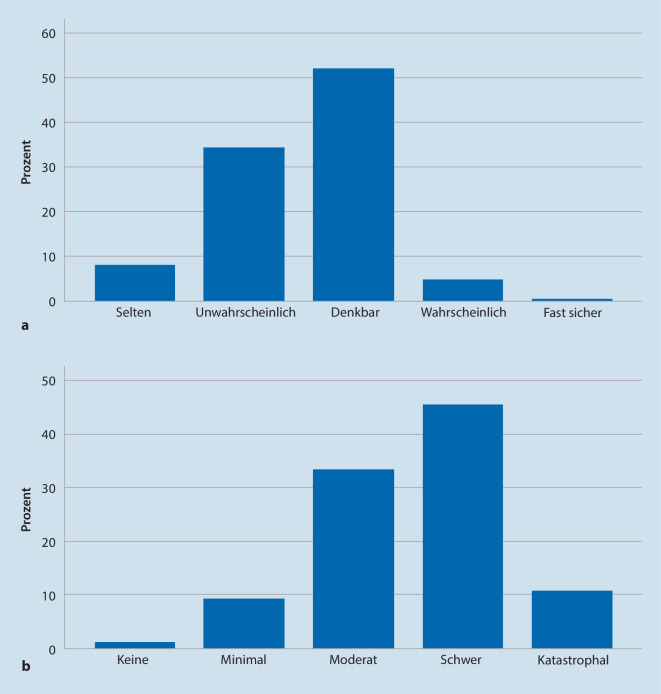

Bei der systematischen Risikobewertung der eingeschlossenen Fälle durch die Autoren anhand der Risikomatrix (≙ Produkt aus Auftretenswahrscheinlichkeit und möglichem Schadensausmaß) ergab sich für die meisten Ereignisse eine eher geringe Auftretenswahrscheinlichkeit (Abb. 2). Mögliche Folgen wurden jedoch in mehr als der Hälfte der Fälle als „schwer“ bis „katastrophal“ eingeordnet (Abb. 2). Bei der Zusammenfassung dieser Risikobewertung in Risikoklassen ergab sich mehrheitlich ein hohes Risiko („extrem hohes“ 7 % [*n* = 20], „hohes“ 62 % [*n* = 177], „moderates“ 25 % [*n* = 68], „niedriges“ Risiko 6 % [*n* = 16]).

## Subanalyse der Risikobewertung mit Fallmerkmalen

Der Vergleich der Risikobewertung anhand des Produkts der beiden Achsen der Risikomatrix mit Beteiligung unterschiedlicher Analgesieverfahren, einer Mehrfachmedikation bzw. der Kombination von Analgesieverfahren oder der Beteiligung des SD zeigte keine signifikanten Unterschiede (Tab. 4 zu Analgesieverfahren; analgetische Mehrfachmedikation vs. keine Mehrfachmedikation: *p* = 0,806, Z = −0,246, Median 9 (6–12) vs. 9 (6–12); Kombination vs. keine Kombination von Analgesieverfahren: *p* = 0,059, Z = −1,886, Median 8 (6–10,5) vs. 9 (6–12); mit SD Beteiligung vs. ohne SD: *p* = 0,919, Z = −0,102, Median 9 (6–12) vs. 9 (6–12)). Ebenso konnten keine Unterschiede in der Risikobewertung von Ereignissen unterschiedlicher übergeordneter Ursachenebene dargestellt werden (Tab. 4). Wirkten mehrere übergeordnete Fehlerursachen ein, zeigte sich eine leicht erhöhte Risikobewertung im Produkt der Achsen der Risikomatrix (nur eine einwirkende übergeordnete Fehlerursache vs. mindestens zwei einwirkende Fehlerursachen, 9 [8–12] vs. 8 [6–12], *p* = 0,038, Z = −2,072).

**Table 4:** 

	Risikoscore (Median (1.–3. Quartil))	Statistik
*Analgesieverfahren*		
Orale Analgetika	8 (5,5–11)	*p* = 0,110, H = 7,548
Parenterale Analgetika	9 (8–12)	
Patientenkontrollierte intravenöse Analgesie („patient controlled intravenous analgesia“; PCIA)	9 (8–12)	
Periphere Regionalanästhesieverfahren	8 (6–12)	
Rückenmarknahe (peridurale) Regionalanästhesieverfahren	9 (6–12)	
*Übergeordnete Ursachenebene*		
Menschliches (Fehl‑)Verhalten: Personal	9 (6–12)	*p* = 0,65, H = 7,220
Menschliches (Fehl‑)Verhalten: Patienten, Angehörige	9 (6–12)	
Technik	6 (6–12)	
Organisation	9 (6–12)	

## Diskussion

Wenn auch stets ein erheblicher Melde- und Auswertungsbias zu berücksichtigen ist, zeigen die Ergebnisse dieser Studie, dass im Rahmen von berichteten Zwischenfällen in der Akutschmerztherapie relevante Risiken für Patienten entstehen können. Die Klassifikation des Risikos, welches sich in den eingeschlossenen Fällen für die Patienten ergab, war mehrheitlich „hoch“. Bei differenzierter Betrachtung zeigte sich, dass dies vorwiegend durch die in der Risikomatrix meist als hoch eingeschätzten potenziellen Konsequenzen getrieben wurde und die Auftretenswahrscheinlichkeit meist weniger hoch war. Menschliche Fehler, Abweichen von der Routine und organisatorische Aspekte standen oft im Zusammenhang mit den Zwischenfällen. Es fanden sich dabei inhaltlich unter den hier eingeschlossenen Fällen zahlreiche in der klinischen Praxis bekannte, potenziell modifizierbare und vermeidbare Probleme, wie beispielsweise die Verwechslung von Anschlüssen und eine daraus resultierende i.v.-Applikation von Lokalanästhetika (z. B. über einen zentralen Venenkatheter), Verwechslung von Wirkstoffen (z. B. Sufentanil anstatt Piritramid im Aufwachraum oder Applikation von Clonidin oder Metronidazol über einen PDK anstatt Ropivacain), Opioidüberdosierungen aufgrund von Fehldosierung bei Austauschpräparat bei nichtlieferbarem Hauspräparat, Fehlmischungen/Fehlkonzentration bei PCIA-Systemen, überhöhte Laufraten, z. B. um Zehnerpotenz, bei Einstellung der PDK-Pumpe, kumulierte Bolusapplikationen bei fehlendem Rückschlagventil und Rückstau in den Infusionsschlauch bei PCIA, aber auch über Tage ausbleibende postoperative Überwachung und Betreuung von periduralen Verfahren bei ausgebliebener Anmeldung von speziellen Analgesieverfahren beim Schmerzdienst.

## Menschliche Fehler und unzureichende Mitarbeiterkompetenzen als Risikoquelle

Wie auch andere Analysen zeigen unsere Ergebnisse, dass menschliches Fehlverhalten mit den meisten der analysierten Fälle im Kontext der Akutschmerztherapie assoziiert ist [35]. Themen, wie Mehrfachbelastung, Leistungsdruck und Zeitnot des Personals, aber auch fehlende Kenntnisse bzw. fehlende Einarbeitung der Mitarbeiter stellen kritische Faktoren dar, die im Rahmen von Ressourcenknappheit und unter ökonomischem Druck der Krankenhäuser eine hohe Relevanz haben. Untersuchungen zeigen einen engen Zusammenhang zwischen Mortalität und Personalausstattung [2, 3]. Die Behandlungsfälle pro Vollkraft im Pflegedienst haben beispielsweise zwischen den Jahren 2003 und 2015 um ca. 10 % zugenommen [4].

Kritische Ereignisse aufgrund fachlicher Fehler, fehlender Kenntnisse und fehlender bzw. unzureichender Einweisung in Geräte sind haftungsrechtlich besonders kritisch zu bewerten und u. a. durch eine umfassende Einarbeitung sowie die vorgeschriebenen Schulungen vermeidbar. Es zeigt auch die Notwendigkeit von regelmäßigen fachlichen Schulungen zum Thema Schmerzmanagement auf. Doch gerade im Kontext von Mehrfachbelastung, Leistungsdruck und Zeitnot zeigt sich in der Praxis oft, dass gerade Schulungen und Einarbeitung von Mitarbeitern mit als Erstes kürzer kommen. Hier stehen Klinik- und Abteilungsleitungen in Verantwortung. Haftungsrechtlich können sie im Rahmen des Organisationsverschuldens belangt werden [10].

Handlungskompetenz der Klinikmitarbeiter hat im Kontext Patientensicherheit einen hohen Stellenwert. Die DGAI hat für ärztliche und pflegerische Mitarbeiter von Schmerzdiensten im Jahr 2019 neben qualifikatorischen Mindestanforderungen auch Kompetenzen definiert, die im Rahmen einer Rotation in den Schmerzdienst durch ärztliche Mitarbeiter erlernt werden sollten [9]. Zu diesen gehören neben medizinischen und rechtlichen Grundlagen, Untersuchungs- und Therapiekompetenzen auch technische und organisatorische Kenntnisse [9]. Die Stärkung der Mitarbeiterkompetenz sollte aus Sicht der Autoren eine hohe Priorität zur Risikoreduktion im Rahmen der Akutschmerztherapie haben. Hier haben standardisierte Einarbeitungskonzepte, „standard operating procedures“ und Notfallalgorithmen einen positiven Einfluss.

## Falsche Applikationswege und Medikamentengaben

Die in unserer Analyse am häufigsten identifizierten Probleme waren falsche Applikationswege, Medikamentenverwechselungen und Dosierungsfehler. Dies entspricht auch anderen Untersuchungen, in denen Medikamenten- und Applikationswegverwechslungen als die häufigsten Problematiken im Kontext der Patientensicherheit identifiziert wurden [21, 22]. Diese lassen sich durch standardisierte Muster und Farbetikettierungen von Spritzen, Infusionsbeuteln, fertigen Medikamenten-Applikation-Bags und liegenden Kathetern reduzieren. Hierzu sollten die Empfehlungen zur Kennzeichnung von Spritzen in der Intensiv- und Notfallmedizin auch in den anderen Versorgungsbereichen des Krankenhauses konsequent genutzt werden [30, 31]. Ebenso wären zahlreiche der gemeldeten Zwischenfälle vermeidbar gewesen, wenn die von der DGAI empfohlenen (und bereits seit Langem als Notwendigkeit erachteten und diskutierten), unterschiedlichen Anschlüsse für parenterale und neuroaxiale Verfahren bereits implementiert worden wären (DGAI-Kommission für Normung und technische Sicherheit: Implementierung der ISO 80369‑6, ein neuer Standard für neurale Konnektoren) [32]. Da die Akutschmerztherapie sich nicht nur auf die anästhesiologischen und intensivmedizinischen Überwachungsbereiche der Krankenhäuser beschränkt, sondern schnittstellenübergreifend auch auf Normalpflegestationen erfolgt, sollten aus Sicht der Autoren diese Standards für das Schmerzmanagement bereichs- und abteilungsübergreifend in den Kliniken gelten und flächendeckend angewandt werden (insbesondere im Kontext regionalanästhesiologischer Verfahren und i.v.-Opioid-Applikation). Weitere Schritte zur Reduktion von Medikamentenfehlern können standardisierte Lagerung, „Order-entry“-Systeme, elektronische Ausgabeschränke oder das Vieraugenprinzip sein [19, 21, 33].

Gerade bei den in der Akutschmerztherapie üblichen Pumpensystemen – insbesondere bei der Opioidapplikation bei der PCIA – sind bis heute manuelle Mischungen zur Füllung des Reservoirs notwendig. Wünschenswert im Sinne der Risikoreduktion wären auch hier fertige Mischungen oder passende Ampullengrößen. Neben den Mischungsverhältnissen ergibt sich hier auch noch ein Risikopotenzial durch falsche Programmierung. Neben technischen Ansätzen, wie vorprogrammierten Behandlungssets oder Sperrung bestimmter Dezimalstellen, sollte organisatorisch die manuelle Füllung und Programmierung möglichst in der Hand einer begrenzten Anzahl qualifizierter Mitarbeiter bleiben.

## Organisation als Risikofaktor

Der große Einfluss organisatorischer Aspekte, wie beispielsweise ungeklärte Verantwortlichkeit, zeigt, wie wichtig die Strukturierung und Prozessorganisation der Akutschmerztherapie in den Kliniken ist. Die Festlegung von Verantwortlichkeiten wird seit vielen Jahren gefordert. Die zuletzt gültige Version der Leitlinie zur Behandlung akuter und posttraumatischer Schmerzen formulierte die Empfehlung, dass übergeordnete Rahmenbedingungen zur Durchführung der perioperativen und posttraumatischen Schmerztherapie zwischen den beteiligten Fachgebieten gemeinsam schriftlich formuliert werden sollen [24]. Eine Mustervereinbarung zur Zusammenarbeit in der Schmerztherapie des chirurgischen und anästhesiologischen Berufsverbandes besteht seit dem Jahr 1992 und gilt in aktualisierter Version aus dem Jahr 2019 [13, 37]. Bundesweite Struktur- und Prozessdaten zeigen jedoch, dass solche schriftlichen Vereinbarungen nicht in allen Krankenhäusern vorliegen, insbesondere nicht in der Versorgung nichtoperativer Patienten [11].

Behandlungspfade und Standards können zudem einen erheblichen Anteil zur Qualitätsverbesserung leisten [12]. Diese sollten ebenfalls klare Vorgaben enthalten, wer wofür verantwortlich ist [6, 7]. Viele der bisweilen in Kliniken implementierten Standards zur Akutschmerztherapie sind inhaltlich und konzeptionell hierzu nicht ausreichend geeignet [6, 7].

## Unzureichende Risiko- und Fehlerkultur in der Akutschmerztherapie?

Hinsichtlich des Umgangs der Kliniken mit Risiken der Akutschmerztherapie ist kritisch zu bewerten, dass in knapp der Hälfte der von uns analysierten Fälle die Problematiken bekannt zu sein scheinen. Trotz dessen, dass hier ein erblicher Melder-Bias besteht, könnten diese Rückmeldungen auf eine ungünstige Risiko- und/oder Fehlerkultur innerhalb der Kliniken bzw. im Themenbereich Schmerzmanagement hinweisen. Der Umgang mit Fehlern und Zwischenfällen in einem Arbeitsbereich oder Versorgungssystem spiegelt das Risikoniveau wider. Dass die Akutschmerztherapie in manchen Krankenhäusern bezüglich des Versorgungsstandards geringer priorisiert wird, zeigt sich in den oft unzureichend vorhandenen personellen und organisatorischen Gegebenheiten bezüglich Überwachung und rechtlichen Anforderungen der Überwachung medikamentöser und invasiver Analgesieverfahren [10]. Einige Kliniken nutzen sowohl periphere als auch rückenmarknahe regionalanästhesiologische Verfahren mit kontinuierlicher Administration und patientengesteuerten Boli, inkl. periduraler Opioidapplikation auf Normalstationen, ohne dass überhaupt ein Schmerzdienst verfügbar bzw. die Ansprechbarkeit eines Schmerzdienstes 24 h am Tag sichergestellt ist [8]. Zu viele Kliniken zeigen (ggf. auch aus wirtschaftlichem Druck oder Personalmangel) bewusste Risikobereitschaft, indem sie Ärzte in der Akutschmerztherapie durch nichtärztliche Mitarbeiter substituieren und diesen auch nichtdelegierbare ärztliche Leistungen überlassen [8, 10]. Oft fehlt zudem eine definierte (oberärztliche) Verantwortung für den Bereich und entsprechende Weiterbildung von Weiterbildungsassistenten in der Akutschmerztherapie [8].

Dies birgt zum einen rechtliche Haftungsrisiken und Organisationsverschulden in sich, aber auch Folgerisiken hinsichtlich fehlender Entwicklung oder Weitergabe von Kompetenzen in der Akutschmerztherapie, welche in der Folge das Patientenrisiko bezüglich Zwischenfällen und Behandlungsfehlern erhöht [10]. Zudem ist bei fehlender organisatorischer Verantwortlichkeit ein unzureichender Umgang mit bestehenden Risiken und auftretenden Zwischenfällen zu erwarten.

## Proaktives Risikomanagement in der Akutschmerztherapie

Im Akutschmerzmanagement kommt dem Schmerzdienst und dem Monitoring eine besondere Bedeutung für die Risikoreduktion der Patienten zu. Eine Umfrage zur Anwendungssicherheit der PCIA zeigte einen engen Zusammenhang zwischen dem Umfang des Monitorings und den wahrgenommenen Komplikationen [5]. Es gilt, Barrieren und Sicherheitsnetzte zum Schutz vor ungünstigen Verkettungen in die Behandlungsprozesse einzubauen, und um ungünstige Verläufe frühzeitig wahrnehmen und dadurch verhindern zu können.

Dass durch eine systemische Herangehensweise auch nachvollziehbar eine Risikoreduktion in der Akutschmerztherapie erfolgen kann, zeigte die Hamilton Acute Pain Safety Study [28]. Unter Einbezug von 35.384 eingeschlossenen Patienten erfolgte nach einer Ausgangserfassung eine Intervention anhand einer systematischen Fehler-Ursachen-Analyse. In der Nachbeobachtung nach Intervention konnte eine Reduktion des Auftretens kritischer Ereignisse im Rahmen der Akutschmerzbehandlung dargestellt werden. Traten vor der Intervention bei jedem 42. Patienten Ereignisse auf, war dies in der Nachbeobachtung nur noch bei jedem 68. Patienten der Fall.

Eine andere Interventionsstudie bezüglich Zwischenfällen der PCIA-Therapie konnte eine Reduktion von Zwischenfällen um 22 % und 72 % weniger Fehlermeldungen erreichen [29].

Der in unserer Untersuchung verwendete Ansatz der Auswertung einer CIRS-Datenbank zeigt eine weitere Option, die Sicherheit des Akutschmerzmanagements, kombiniert mit einer Fehler-Ursachen-Analyse, zu optimieren.

## Limitation

Wie bei allen retrospektiven Datenanalysen sind auch bei dieser Untersuchung einige Limitationen inhärent. Die Analyse vermag keine Aussagen zu machen, über die Grundgesamtheit von Zwischenfällen in der Akutschmerztherapie. Da die Auswertung anhand einer Sammlung berichteter Zwischenfälle eines bundesweiten CIRS-Meldesystems erfolgte, besteht selbstverständlich auch keine inhaltliche Generalisierbarkeit von Häufigkeit und Schweregrad der aufgetretenen kritischen Ereignisse, und alle Aussagen können sich nur auf die Gesamtmenge der eingeschlossenen Fallberichte beziehen.

Es handelt sich um ein Meldesystem, mehrheitlich geprägt von Fachvertretern, sodass davon auszugehen ist, dass hier ggf. verstärkt fach- bzw. berufspolitische Themen tangierende Ereignisse gemeldet werden. Ein Hinweis hierfür ist der hohe Anteil an Fällen mit Einbezug des Schmerzdienstes.

Für die wissenschaftliche Analyse der einzelnen CIRS-Reports besteht ein sehr begrenztes Kontextwissen, welches sowohl bezüglich Umfang als auch inhaltlich von der Ausführlichkeit und Detailtreue der freien Fallbeschreibung der Melder abhängig ist. Somit ist aufgrund der subjektiven Schilderungen zudem stets ein Auswertungsbias, der sowohl stark geprägt von der Sichtweise des Melders als auch der Interpretation der Analysierenden sein kann, zu berücksichtigen. Aus dem unklaren Meldeverhalten in einem solchen freiwilligen Fehlermeldesystem kann sich nicht nur eine zufällige, nichtrepräsentative, möglicherweise negativ selektierte Sammlung von Zwischenfällen ergeben. Neben der Subjektivität der Schilderung enthalten solche Meldungen oft auch einen gewissen Anteil indirekter Beschwerden durch Mitarbeiter. Dieser letzte Aspekt vermag in einem bundesweiten Meldesystem wie CIRS-AINS zwar ggf. etwas geringer zum Tragen kommen, da die Meldungen nicht betriebsintern Folgen für Vorgesetzte oder die interne Organisation haben. Er ist aber dennoch als Limitation zu berücksichtigen.

Limitationen durch begrenztes Kontextwissen und Detailtreue inhaltlicher Ausführungen haben wir versucht zu reduzieren, indem wir eine mehrstufige Auswertung, inklusive Reevaluation im Team von unterschiedlich eingeschätzten Fällen, vorgenommen haben. Durch weitgehend dichotome Auswertung (im Sinne von „Merkmal vorhanden“ vs. „nichtvorhanden“) haben wir zudem versucht, den Melder-Bias zu reduzieren. Dies ist jedoch dadurch limitiert, dass andere Faktoren vorhanden sein können, aber nicht erfasst wurden. Eine retrospektive Analyse kann keine Kausalität herstellen, was die Einschätzung von fallindividueller Patientenbelastung, Patientengefährdung sowie des Risikopotenzials eines bestimmten Verfahrens in ihrer Aussagekraft einschränkt. Unsere Untersuchung kann auch methodologisch keine definitiven Aussagen zu Risiken bestimmter Analgesieverfahren machen. Die Einschätzung des Risikos der Zwischenfälle bezieht sich nicht auf das medizinische Risiko eines speziellen Analgesieverfahren. Bei den analysierten Zwischenfällen geht es um Situationen und Einflusskonstellationen, die sich rund um diese Verfahren ereignen. Die Quantifizierung des Risikos hat dabei das Ziel darzustellen, welche Ursachen/Ereignisse im Kontext des klinischen Risikomanagements niedrige oder hohe Risikopotenziale aufweisen, um diese zu steuern, also z. B. zu stratifizieren, um Prioritäten bei Veränderungsprozessen zu setzen.

## Schlussfolgerung bzw. „Fazit für die Praxis“

Zwischenfälle in der Akutschmerztherapie können hohe Risiken für die Patienten in sich bergen. In dieser Untersuchung von CIRS-Berichten standen Zwischenfälle oder Beinahezwischenfälle mehrheitlich im Zusammenhang mit Fehlern und fehlenden Kompetenzen des Personals, oft aus Zeitdruck und Arbeitsbelastung, sowie aufgrund unzureichender Organisation. Die Akutschmerztherapie ist schon aufgrund zahlreicher Schnittstellen ein Versorgungsbereich mit erheblichen organisatorischen Risiken. Daher sollten zur Risikominimierung und zur Verbesserung der Patientensicherheit Behandlungsprozesse und Verantwortlichkeiten abteilungs- und bereichsübergreifend definiert werden sowie notwendige personelle Ressourcen zur Verfügung stehen.

Außerdem sollte Risikomanagement fester Bestandteil des klinikinternen Schmerzmanagements sein, um Behandlungsrisiken entgegenzuwirken bzw. Fehler und Zwischenfälle als Quellen für Veränderungsprozesse nutzen zu können.

### Infobox 1 Themen der Fälle, in denen anhand der zur Verfügung stehenden Informationen der Fallkonstellation ein „Patientenschaden“ angenommen werden musste (ein unerwünschtes Ereignis, dass eine relevante therapeutische Intervention notwendig machte bzw. mit zusätzlicher Belastung durch therapeutische Folgen für den Patienten verbunden war, die über das unmittelbare Fallgeschehen andauern), *n* = 27

- Analgetikumbolus über Katecholaminschenkel des zentralen Venenkatheters appliziert (hypertensive Entgleisung mit Nachblutung)
- Opioidanalgetikum bei Kind 3‑fach überdosiert (Beatmung)
- Intravasal liegender PDK (Reanimation)
- Applikation von Kaliumchlorid über den PDK (passagere Tetraplegie, Spastik, metabolische Acidose)
- Verwechselung von Oxycodon mit Mivacurium (Beatmung und Sedierung)
- Intravasal liegender Ischiadikuskatheter (Asystolie, Reanimation)
- Dosisverwechselung auf 5‑fach erhöhte Opioiddosis (Hypoxämie, Antagonisierung mit Naloxon, anschließend „nur“ Überwachung auf der Normalstation ohne Monitoring – es folgten Rebound und Notwenigkeit zur Beatmung)
- Opioidüberdosierung bei Liefermangel des Hauspräparates und Dosisverwechslung des Austauschpräparates (Bewusstseinsverlust und Hypoxämie mit Verdacht auf einen hypoxischen Hirnschaden)
- Verwechslung von Piritramid mit Sufentanil (Apnoe und Beatmung)
- Patient erhielt auf der Normalstation Piritramid und Lorazepam (es folgten Sedierung und Hypoxämie und Hirnstamminfarkt)
- Bei PDK-Anlage schert der Katheter ab und ein Stück verbleibt paravertebral (operative Entfernung)
- Durchtrennung PDK bei Anlage und Tunnelung (operative Entfernung)
- Abriss des PDK beim Entfernen und verbleibt in situ (operative Entfernung)
- Schmerzkatheteranlage für Gelenkeingriff auf falscher Seite, bei Anlage in Vollnarkose
- Anlage des peripheren Schmerzkatheters auf falscher Seite
- Verwechslung von Piritramid mit Sufentanil (Reanimation)
- Duraperforation mit nachfolgenden Kopfschmerzen bei schwer gängigem Spritzenstempel
- Asystolie bei PDK und zusätzlich Neostigmin (Reanimation)
- Verwechslung von NaCl zum Spülen mit Piritramidspritze nach Bolusgabe des Piritramids bei Kleinkind (Apnoe mit Beatmung und Antagonisierung)
- Katheter bildete „Knoten“ in situ und ließ sich nicht mehr ziehen (operative Entfernung)
- Anlage des peripheren Schmerzkatheters auf falscher Seite
- Umstellung der Analgesie auf transdermales Fentanyl bei multimorbidem Patienten, Applikation erfolgte 10-fach erhöht zur eigentlich beabsichtigter Dosierung (Apnoe und Beatmung)
- Hautschaden durch zu enge Thrombosestrümpfe bei fehlende Sensibilität unter PDK-Therapie
- Paracetamolapplikation bei bekannter Allergie (schwere allergische Reaktion)
- intravasale „peridurale“ Sufenta-Applikation (Bewusstlosigkeit und Apnoe, Beatmung und Antagonisierung)
- Applikation von Metamizol bei bekannter Allergie (schwere allergische Reaktion)
- Verwechslung von Ampullen mit Sufentanil unterschiedlicher Konzentration (50 µg/ml statt 5 µg/ml) nach Wechsel des Herstellers (Apnoe, Beatmung und Antagonisierung)

## Footnotes

333 Unerwünschtes Ereignis, das im beschriebenen Fallgeschehen mindestens eine Vitalfunktion beeinträchtigte.

444 Unerwünschtes Ereignis, das eine relevante therapeutische Intervention notwendig machte bzw. mit zusätzlicher Belastung durch therapeutische Folgen für den Patienten verbunden war, die über das unmittelbare Fallgeschehen andauern.
